# Study on the Absorbed Fingerprint-Efficacy of Yuanhu Zhitong Tablet Based on Chemical Analysis, Vasorelaxation Evaluation and Data Mining

**DOI:** 10.1371/journal.pone.0081135

**Published:** 2013-12-10

**Authors:** Haiyu Xu, Ke Li, Yanjun Chen, Yingchun Zhang, Shihuan Tang, Shanshan Wang, Dan Shen, Xuguang Wang, Yun Lei, Defeng Li, Yi Zhang, Lan Jin, Hongjun Yang, Luqi Huang

**Affiliations:** 1 Institute of Chinese Materia Medica, China Academy of Chinese Medical Sciences, Beijing, P.R. China; 2 Shandong Provincial Key Laboratory of Automotive Electronic Technology, Institute of Automation, Shandong Academy of Sciences, Jinan, P.R. China; 3 National Resource Center for Chinese Materia Medica, China Academy of Chinese Medical Sciences, Beijing, P.R. China; 4 Analysis and Test Center, Shandong Academy of Sciences, Jinan, PR China; 5 Laboratory of Drug Metabolism and Pharmacokinetics, Shenyang Pharmaceutical University, Shenyang, PR China; 6 National Glycoengineering Research Center, Shandong University, Jinan, PR China; Macau University of Science and Technology, Macau

## Abstract

Yuanhu Zhitong Tablet (YZT) is an example of a typical and relatively simple clinical herb formula that is widely used in clinics. It is generally believed that YZT play a therapeutical effect *in vivo* by the synergism of multiple constituents. Thus, it is necessary to build the relationship between the absorbed fingerprints and bioactivity so as to ensure the quality, safety and efficacy. In this study, a new combinative method, an intestinal absorption test coupled with a vasorelaxation bioactivity experiment *in vitro*, was a simple, sensitive, and feasible technique to study on the absorbed fingerprint-efficacy of YZT based on chemical analysis, vasorelaxation evaluation and data mining. As part of this method, an everted intestinal sac method was performed to determine the intestinal absorption of YZT solutions. YZT were dissolved in solution (n = 12), and the portion of the solution that was absorbed into intestinal sacs was analyzed using rapid-resolution liquid chromatography coupled with quadruple time-of-flight mass spectrometry (RRLC-Q-TOF/MS). Semi-quantitative analysis indicated the presence of 34 compounds. The effect of the intestinally absorbed solution on vasorelaxation of rat aortic rings with endothelium attached was then evaluated *in vitr*o. The results showed that samples grouped by HCA from chemical profiles have similar bioactivity while samples in different groups displayed very different. Moreover, it established a relationship between the absorbed fingerprints and their bioactivity to identify important components by grey relational analysis, which could predict bioactive values based on chemical profiles and provide an evidence for the quantification of multi-constituents.

## Introduction

The Chinese materia medica (CMM) is a unique healthcare system that has been successfully applied for thousands of years in East Asian countries, such as China, Japan, and Korea. Recently, the CMM has attracted increasing global attention due to their long history of clinical application, reliable therapeutic effect, and low toxicity. However, the complexity of CMMs provides a significant challenge for researchers to seek enough scientific evidences to support their efficacy [1]. Generally, most of CMMs are taken orally and the absorbed constituents are more likely to play a role in the therapeutic action *in vivo*. Thus, it is necessary to obtain the absorbed chemical profiles, evaluate their bioactivities and build the relationship between absorbed peak and bioactivity so as to identify the bioactive compositions [2] and ensure the efficacy of the CMM products [3]. But to solve the above problems, the three key techniques are as follows: firstly, a method with high-throughput and sensitivity is needed to analyze chemical constituents as more as possible. Rapid resolution liquid chromatography coupled with quadruple time-of-flight mass spectrometry (RRLC-Q-TOF/MS) has short analytical time and high sensitivity for identification of both known and unknown, macro and micro compounds as well [4]. Secondly, a combinative bioassay that consists of an intestinal absorption test coupled with an *in vitro* bioactivity experiment is a simple, sensitive, and feasible method for evaluating CMM formulations, which allows researchers to exclude unabsorbed constituents that might produce false positive or negative pharmacological actions in bioassays and shows high sensitivity which can exhibit different activities due to the changing contents of the compounds in CMMs [5]. The third technique is data mining, with its main aim to discover hidden patterns from large data sets and to summarize them into useful information through analysis of the data from different perspectives, including statistics and artificial intelligence [6].

Yuanhu Zhitong Tablet (YZT) is an example of typical and relatively simple clinical herb formula. It contains *Angelicae dahuricae Radix* and *Corydalis Rhizoma* and is widely used in the treatment of gastralgia, costalgia, headache, and dysmenorrhea caused by qi stagnancy and blood stasis [7]. The naturally abundant compounds in these two plants have been chemically isolated and identified. Bioassays indicate that alkaloids and coumarins are the main active constituents of these plants and some of them have multiple pharmacological activities, including anti-nociceptive [8], anti-inflammatory [9], anxiolytic [10], spasmolytic [11], and vasorelaxation activities [5]. Vasorelaxation is very important for the treatment of pain [12], [13]. In the CMM theories, “it is the obstruction that causes the pain” and many factors can lead to migraine, such as disorders of blood vessel function, blood vessel convulsion [14], [15]. In that case, migraine can be relieved by dredging vessels. Practitioners of Western medicine (WM) also reckon that vasospasms cause headaches (migraine) [16] and dysmenorrhea [17].

In recent years, our research teams have carried out consistent studies in an attempt to understand action mechanisms of YZT. Firstly, the chemical fingerprint and quantitative analysis of multiple constituents were applied to the quality control of YZT [18], [19]. Next, an everted intestinal sac method was used to determine the components of YZT that were absorbed by the intestine [20]. These studies helped to identify the active components of YZT to further elucidate their pharmacological mechanisms. Absorption solutions of YZT obtained via using the everted sac method were full of vasorelaxant substances that produced a dose–response relationship. Moreover, the activity of intestinally absorbed YZT solution was much higher than that of one or a few dominant chemical components [5]. However, it was unclear whether the innovative combinative method of bioassay could be applied to appraise the quality of the different YZT samples and bridge the relationship between constituents and biactivity (RCB) and identified key components (KC) by data mining that relate to vasorelaxation activity. In this study, in order to better conduct the quality control of YZT products, we used the combinative method that involved an intestinal absorption test coupled with a vasorelaxation evaluation. Twelve batches of intestinally absorbed YZT solution were analyzed via RRLC-Q-TOF. Then, the RCB and KC of YZT were identified by data mining and validated by further experiments.

## Experimental

### 1. Materials and reagents

HPLC-grade acetonitrile was obtained from Tedia (Fairfield, OH, USA). Formic acid was of analytical grade and obtained from Shanghai Chemical Regent Co. (Shanghai, China). Water was purified using a Milli-Q system (Millipore, Billerica, MA, USA). Twelve batches of YZT samples that produced by different manufacturing factories were purchased from local drug stores (**Table 1**.).

**Table 1:** A summary of the tested samples of YZT.

Sample No.	Pharmaceutical factory	Batch No.	Production date
S01	Foci, Shanxi	11B1	2011.6.25
S02	Foci, Shanxi	11D3	2011.7.20
S03	Banzhou Tianlong, Guangxi	100501	2010.5.8
S04	Banzhou Tianlong, Guangxi	091201	2009.12.30
05	Shuzhong, Sichuan	091203	2009.12.17
S06	Shuzhong, Sichuan	100601	2010.6.3
S07	Shibiao, Guangxi	100105	2010.1.8
S08	Geruilin,Chongqing	091201	2009.12.4
S09	Foshan Dezhong, Guangdong	10004	2010.4.29
S10	Foshan Dezhong, Guangdong	10008	2010.9.14
S11	Foci, Shanxi	11D4	2011.7.25
S12	Shuzhong, Sichuan	100502	2010.5.14

Imperatorin, isoimperatorin, osthole, protopine, berberine, palmatine, bergapten, psoralen and tetrahydropalmatine standards were purchased from the National Institute for the Control of Pharmaceutical and Biological Products (Beijing, China). Byakangelicol, byakangelicin and coptisine were purchased from Chengdu Herbpurify Co. (Chengdu, China). Xanthotoxin, oxypeucedanin, and tetrahydroberberine were obtained from Shanghai Winherb Co. (Shanghai, China). Corydaline was supplied by Sigma Co. (Sigma, USA). Pimpinellin was from Shanghai Touto Biotech. (Shanghai, China). A-allocryptopine was purchased from Shenzhen Meihe Biotech. (Shenzhen, China). All standards were at least 98% pure and were suitable for RRLC-Q-TOF-MS analysis. Ligustrazine was purchased from Nanjing Qingze Pharmaceutical Technology Co., Ltd (Nanjing, China).

### 2. Sample solution preparation

The coating was removed from all samples and the remaining portion of each sample was powdered and sieved via 60 mesh. Then each sample equal to 24 g crude drug was then weighed precisely, and was extracted under reflux with 240 mL of 70% ethanol for 1 h. The extract was concentrated under reduced pressure using a rotary evaporator at 70°C, followed by dilution with Tyrode buffer (mmol/L; NaCl 136.75, KCl 3.76, NaHCO_3_ 11.90, NaH_2_PO_4_ 0.42, MgCl_2_ 1.05, CaCl_2_ 1.80, and glucose 5.56, pH 7.4) to a concentration of 0.16 g/ml. At the same time, α-allocryptopine, protopine and ligustrazine (a positive control) were directly diluted in the Tyrode buffer with the concentration of 0.58, 0.56 and 0.50 mg/ml, respectively.

### 3. Animals

Adult male Sprague-Dawley rats weighing 240±10 g (from the Experimental Animal Center of Peking University Health Science Center, Beijing, China) were used for everted intestinal sac experiments and isolated vascular ring models. The animal welfare and experimental procedures complied with the Guide for the Care and Use of Laboratory Animals (National Research Council of the USA, 1996) and related ethical regulations of the China Academy of Chinese Medical Sciences. The protocols were approved by the Animal Ethics Committee of Laboratory Animals at the Institute of Laboratory Animal Resources of Beijing (Beijing, China).

### 4. Preparation of intestinally absorbed solution

Everted sacs were prepared according to the modification of the procedure described previously [20]. Briefly speaking, rat was fasted for 12 h before the experiment. Under anesthesia, the abdomen was dissected along the median line and the linea alba. Then, the jejunum and ileum were immediately removed and rinsed in ice-cold Tyrode buffer solution. Four fourteen-centimeter segments of intestine were isolated to perform the intestinal absorption study. The intestinal segments were everted using a soft silica gel tube, and then the sacs were washed with the buffer for three times, and finally each segment was ligated with cotton thread at both ends to form a sac, in which Tyrode buffer solution was filled. The filled sac was incubated in Magnus' bath with Tyrode buffer for 5 min for equilibration, and after that the buffer was replaced with YZT solution or Ligustrazine solution. During the 2 h incubation period, the solution was maintained at 37°C and continuously aerated with O_2_/CO_2_ (95%/5%). After incubation, the sacs were removed and blotted dry using gauze. The serosal-side solutions, which contained absorbed constituents, were drained into small tubes. The intestinally absorbed solutions and blank Tyrode buffer were stored at −20°C for further analysis. Samples were prepared for RRLC-ESI-Q-TOF analysis by drying 100 µL of each solution at 37°C under a stream of nitrogen. The residue was dissolved in 100 µL of methanol, and the samples were centrifuged at 12000× *g* for 10 min. Then the supernatant was directly injected into the RRLC-ESI-Q-TOF. The remainder of each sample was used to evaluate the vasorelaxation activity of YZT *in vitro*.

### 5. RRLC-ESI-Q-TOF instrument, chromatographic conditions, and mass spectrometry conditions

Chromatographic experiments were performed using an Agilent 1200 RRLC System (Agilent Crop., Santa Clara, CA, USA) equipped with a quaternary pump, an online vacuum degasser, an auto-sample injector, and an automatic thermostatic column oven. The mobile phases were as follows: solvent A, 0.2% aqueous formic acid and solvent B, acetonitrile. Gradient elution was as follows: 0–6.0 min, 20–40% B; 6.0–8.0 min, 40–80% B; 8.0–13.0 min, 80% B; 13.0–14.0 min, 80–20% B; 14.0–15.0 min, 20% B. Elution was performed at a flow rate of 0.50 ml/min. The column was an Agilent XDB C18 maintained at 30°C, and the injection volume was 5 µl.

The TOF-MS instrument was an Agilent 6520 quadrupole TOF-MS that was equipped with an electrospray ionization source (ESI). Ionization was performed in the positive electrospray mode. Based on the best response for most compounds, the final parameters were as follows: fragmentor (150 V), Vcap (3500 V), nebulizer (30 psi), drying gas (N_2_, 10 L/min, 350°C). The TOF-MS was calibrated daily, according to the manufacturer's recommendations. The testing mass range was set at m/z 50–1000 with a scanning rate of 2 s^−1^. Reference masses at m/z 121.05087 (purine) and m/z 922.00980 [hexakis (1H, 1H, 3H-tetrafluoropropoxy)-phosphazine] (Agilent Corp.) were continually introduced along with the RRLC stream for accurate mass calibration. The collision energy for each compound varied according to the following formula: [5×(mass/100)]+5. For example, the collision energy of an ion with nominal m/z of 300 would be 20 V.

### 6. Hierarchical cluster analysis of intestinally absorbed YZT solutions

To evaluate the chemical variation of intestinally absorbed YZT solutions, hierarchical cluster analysis (HCA) was performed based on the characteristics of the peak areas in the profiles of the 34 constituent compounds. The peak areas of samples 1–12 formed a 12×34 matrix. Distances between the 12 samples were calculated using SPSS software 18.0 (SPSS Inc. USA).

### 7. Evaluation of vasorelaxation activity in vitro [5]

#### 7.1. Preparation of thoracic aorta rings

After rats were killed by cervical dislocation, the thoracic aorta was carefully removed from each rat and immediately placed into ice-cold Krebs' solution (NaCl 118.96, KCl 4.73, KH_2_PO_4_ 1.17, MgSO_4_ 1.17, NaHCO_3_ 25.0, CaCl_2_ and 2.54, glucose 11.1 mmol/L; pH 7.4). Arterial vessels were carefully prepared by removing connective fat and tissue and cut into 3 to 4 mm-long segments. The segments were mounted in an organ bath containing Krebs' solution using 2 L-shaped stainless steel wire hooks that were inserted into the lumen. The Krebs' solution was maintained at 37°C and O_2_/CO_2_ (95%/5%) and was continually bubbled through the solution. After incubation with no tension for 30 min, the ring segments were allowed to equilibrate for 1 h at a resting tension of 1 g and they were washed every 20 min during the period. Changes in tension were recorded by isometric transducers that were connected to a data acquisition system (Shanghai Alcott Biotech Co., LTD. Shanghai, China).

#### 7.2. Bioactivity evaluation of intestinally absorbed YZT solutions on rat aortic rings with endothelium

After the equilibration, the presence of endothelium was confirmed by inducing relaxation using acetylcholine (ACh, 10^−5^ mol/L) in aortic rings that had been precontracted using phenylephrine (PE, 10^−6^ mol/L). Only tissues with satisfactory endothelium activity (relaxation values, >60%) were used in the experiments. All rings were exposed repeatedly to KCl solution (60 mmol/L) until the tension stabilized and the most contractive degree was set to 100% of relaxation in this condition. Six cumulative doses of intestinally absorbed YZT solution were then added directly to the organ bath. Doses of 50, 100, 200, 400, 800 and 1600 µl were cumulatively added at 15 min intervals. Finally, the percentage ratio of the relaxation was calculated by comparing the contractive degree using 1600 µl YZT solution to the most contractive degree using KCl solution (60 mmol/L). A total of twelve intestinally absorbed samples of YZT solution, the blank intestinally absorbed Tyrode buffer solutions and the Ligustrazine solution were evaluated. The experiment was repeated 8 times. Aortic rings were obtained from a new rat for each replication. Results of bioassay experiments were represented as mean ± S.D. The mean relaxation values from 10 samples at a dosage of 1600 µl were used to evaluate the relationship between solution constituents and bioactivity.

### 8. Grey relational analysis (GRA)

GRA was proposed by Deng [6] in the 1980s and is an important method of grey system theory which has been successfully applied to solve many concrete real-world problems that have complicated interrelationships between multiple variables [21].

#### 8.1. Principles of GRA

The word “grey” in “grey system” indicates a position between “black” (extremely unknown information) and “white” (totally explicit information) and represents information that is incomplete, uncertain, or poor. In other words, a grey system is a mixture of known and unknown information. In the real world, most systems are grey rather than white or black.

GRA is based on the degree of similarity or difference in development trends between an alternative and the ideal alternative. If the trend of differences between the alternative and the ideal alternative is consistent, the 2 alternatives are strongly related [22], [23]. The distinguishing feature of GRA is that small data sets can be handled easily, and the quantitative and qualitative relationships between variables can be identified from numerous factors when there is insufficient information [24].

#### 8.2. GRA Procedure

The GRA calculation process is as follows:

*Step 1. Date processing*

Like general statistical analysis methods, GRA first calls for the standardization of raw data to remove anomalies associated with different measurement units and scales by employing various techniques. This ensures that each raw data series has influence of the same degree on the dependent variables. We used average value processing, as what is shown in the following equation (Eq. 1.) to make the raw data dimensionless.
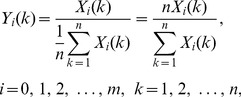
(1)where 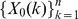 represents the reference sequence (ideal target sequence, i.e., the optimal performance that can be achieved by any of the comparability sequences), and 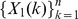, 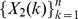, 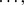 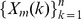 represent the comparability sequences. For convenience of presentation, we denote 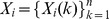 and 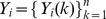 as 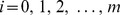.

*Step 2. Grey relational grade (GRG) calculation*

GRG represents the level of correlation between the reference sequence 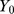 and the comparability sequence 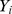, 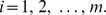 The GRG is used to describe the degree of influence that the comparability sequence exerts over the reference sequence. The GRG increases with the relative importance of a comparability sequence to the reference sequence.

GRG can be calculated as:
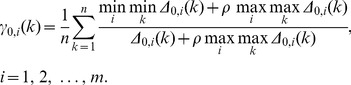
(2)Here 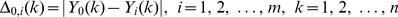. 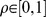 is a distinguishing coefficient, and a value of 0.5 is used in most situations. Generally, the following criteria are used, 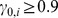 indicates a marked influence, 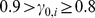 indicates a relatively high influence, 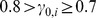 indicates a noticeable influence,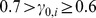 indicates a small influence, and 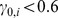 indicates a negligible influence.

*Step 3. Relational polarity analysis*

While GRG indicates how close 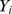 is to 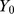 (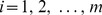), it does not imply that the two have either a positive or negative relationship. We can solve this problem by calculating 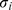, as in Eq. 3.
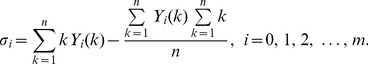
(3)If 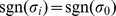 then 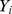 and 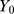 have a positive relationship. If 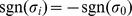 then 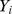 and 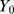 have a negative relationship. Here, the signum function 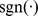 is defined as follows:
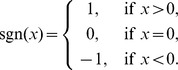



## Results and Discussion

### 1. Optimization of the extraction conditions

In order to optimize the extraction conditions, the effect of extraction method, solvent, and time on extract efficiency were investigated based on the relaxation value obtained using the combinative method of intestinal absorption coupled with vasorelaxation evaluation. Refluxing extraction was more effective than ultrasonic extraction. Further, regarding the tested ethanol concentrations, 70% ethanol was the most efficient extraction solvent. The effect of extraction time on the efficiency of refluxing extraction was investigated at 0.5, 1, 1.5 and 2 h. Finally, it was founded, at an extraction time of 1 h, the highest activity with the lowest solvent dosage could be obtained.

### 2. Optimization of the chromatographic and mass spectrometric conditions

In order to obtain good chromatographic behavior and appropriate ionization, 4 mobile phase systems of methanol-water, acetonitrile-water, methanol-acid aqueous solution, and acetonitrile-acid aqueous solution were compared. The acetonitrile-acid aqueous solution performed better than the others. Formic acid was added into the mobile phase to improve the peak shape and restrain peak tailing, especially those of alkaloids. This was also helpful for evaluating ion response and improving the resolution of these components. The optimal solution was acetonitrile-0.2% aqueous formic acid. MS spectra were studied in both positive and negative modes. The positive mode was applied because better ion-response sensitivity was shown than in the negative mode.

### 3. Analytical method validation

Six independent intestinally absorbed YZT solution samples (S6 was randomly selected) were prepared and analyzed in parallel using the above-established method to evaluate the repeatability of the method. The relative standard deviations (RSDs) of the relative retention times (RRTs) and relative peak areas (RPAs) of the common peaks were less than 1.15% and 4.78%, respectively. The precision of the method was assessed by analyzing the same sample 6 consecutive times within the same day. The RSDs of the RRTs and RPAs of the common peaks were less than 3.23% and 4.65%, respectively. Sample stability was evaluated using the same sample after 0, 4, 8, 24 and 48 h in the autosampler. The RSD of the RRTs and RPAs were less than 5%, illustrating good stability of samples in methanol over the tested period. These results indicate that the method is reliable and applicable for analysis of intestinally absorbed solutions of YZT.

### 4 Chemical analysis of the intestinally absorbed solutions of YZT samples

**Figure 1:**
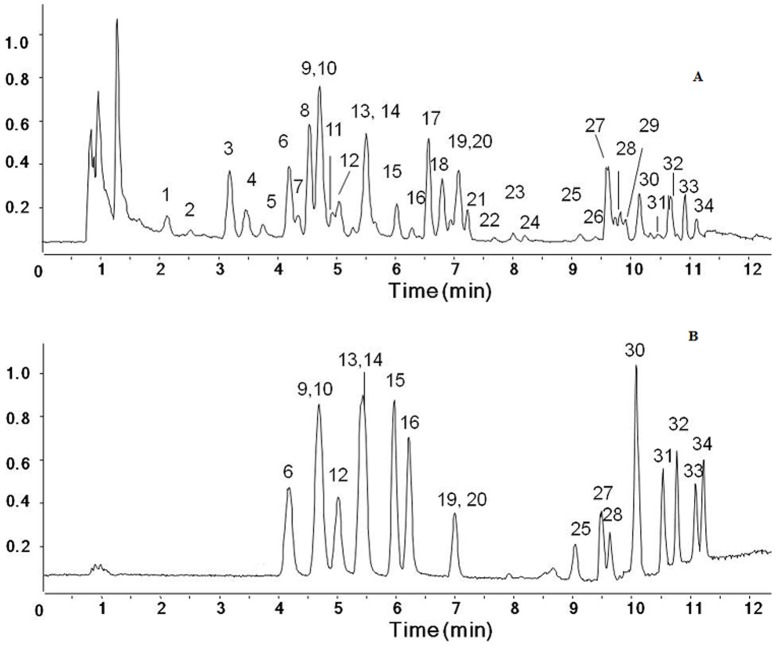
RRLC-ESI-Q/TOF chromatograms (TIC) on positive ion mode. (A) the intestinally absorbed YZT solutions; (B) 18 standard compounds. Peaks: 2, thalicmidine/lirioferine; 3, thalicmidine/lirioferine; 4, Tertiary alkaloid; 5, Tertiary alkaloid; 6, protopine; 9, dl-tetrahydropalmatine; 10, α-allocryptopine; 11, isoboldine; 12, coptisine; 13, tetrahydroberberine; 14, corydaline; 15, palmatine; 16, berberine; 17, dehydrocorydaline; 18, unknown; 19, byakangelicin; 20, byakangelicol; 25, xanthotoxin; 27, pimpinellin; 28, bergapten; 30, oxypeucedanin; 31, psoralen; 32, imperatorin; 33, osthole; 34, isoimperatorin; the others, unknown.

RRLC-ESI-Q/TOF) total ion chromatogram (TIC) profiles of Yuanhu Zhitong tablets and 18 standard compounds are shown in **Figure 1**. Thirty-four compounds were detected and identified based on retention time and MS information, as shown in **Table 2**. Peak areas of 34 compounds of 12 intestinally absorbed YZT samples from various pharmaceutical factories, including different batches from the same manufacturer, are summarized in **Table 3**.

**Table 2:** The retention time (RT), MS data of the 34 common constituents screened out from the YZT by RRLC-Q-TOF.

Peak	RT (min)	Experimental mass (m/z)	Calculated mass (m/z)	Formula	MS/MS (m/z)	Identification compound
1	2.202	314.1747	314.1745	C_13_H_23_O_4_N_5_	283.1320 [M-CH_2_-OH]^+^107.0495 [M-C_10_H_15_N_4_O]^+^	unknown
2	2.540	342.1699	342.1694	C_14_H_23_O_5_N_5_	192.1013 [M-C_9_H_10_O_2_]^+^178.0854 [M-C_10_H_12_O]^+^	thalicmidine/lirioferine [21]
3	3.198	342.1694	342.1694	C_14_H_23_O_5_N_5_	279.101 [M-C_2_H_9_NO]^+^178.0852 [M-C_10_H_12_O_2_]^+^	thalicmidine/lirioferine [21]
4	3.552	356.1853	356.1850	C_15_H_25_O_5_N_5_	192.1009 [M-C_10_H_12_O_2_]^+^	Tertiary alkaloid [22]
5	3.802	356.1852	356.1850	C_15_H_25_O_5_N_5_	192.1013 [M-C_10_H_12_O_2_]^+^	Tertiary alkaloid [22]
6	4.206	354.1339	354.1336	C_20_H_19_O_5_	206.0804 [RDA-tetrahydroisoquinoline]^+^149.0596 [RDA-benzene-ring]^+^	protopine
7	4.299	414.1912	414.1918	C_18_H_23_O_3_N_9_	368.1465 [M-C_2_H_6_O]^+^165.0898 [M-C_14_H_11_N_5_]^+^	unknown
8	4.474	325.1435	325.1442	C_15_H_16_ON_8_	310.1182 [M-CH_3_]^+^294.1238 [M-CH_3_O]^+^279.0999 [M-C_2_H_6_O]^+^	unknown
9	4.702	356.1860	356.1856	C_21_H_25_O_4_N	192.1005 [RDA-tetrahydroisoquinoline]^+^177.0789 [[RDA-benzene-ring]^+^	dl-tetrahydropalmatine
10	4.729	370.1651	370.1649	C_21_H_23_NO_5_	352.0931 [M-H_2_O]^+^206.0795 [RDA-tetrahydroisoquinoline]^+^165.0914 [RDA-benzene-ring]^+^	α-allocryptopine
11	4.912	328.1885	328.1465	C_19_H_21_NO_4_	121.0638 [M-C_15_H_11_O]^+^	isoboldine
12	5.045	320.0920	320.0917	C_19_H_14_NO_4_	292.0922 [M-CO]^+^262.0866 [M-2CO-2H]^+^	coptisine
13	5.419	340.1541	340.1543	C_20_H_21_O_4_N	176.0706 [RDA-benzene-ring]^+^	tetrahydroberberine
14	5.495	370.2010	370.2013	C_22_H_27_NO_4_	207.1205 [RDA-tetrahydroisoquinoline]^+^191.0897 [RDA-benzene-ring]^+^	corydaline
15	5.995	352.1543	352.1543	C_21_H_22_NO_4_	337.1272 [M-CH_3_]^+^322.1078 [M-2CH_3_]^+^308.1268 [M-CH_3_-H-CO]^+^	palmatine
16	6.248	336.1228	336.1230	C_20_H_18_NO_4_	321.0995 [M-CH_3_]^+^292.0944 [M-CH_3_-H-CO]^+^	berberine
17	6.457	366.1717	366.1700	C_22_H_24_NO_4_	351.1445 [M-CH_3_]^+^336.1182 [M-2CH_3_]^+^322.1420 [M-CH_3_-H-CO]^+^308.1298[M-2CH_3_-CO]^+^	dehydrocorydaline [24]
18	6.764	305.1023	305.1027	C_11_H_12_O_3_N_8_	203.0335[M-C_5_H_10_O_2_]^+^	unknown
19	7.025	357.0953	335.1125	C_17_H_18_O_7_	231.0156 [M–H_2_O-C_5_H_8_O]^+^203.0348 [M–H_2_O-C_5_H_8_O-CO]^+^	byakangelicin
20	7.025	317.1019	317.1020	C_17_H_16_O_6_	287.0879 [M-CH_2_O]^+^203.0338 [M-CH_2_O-C_5_H_8_O]^+^175.0402 [M-CH_2_O-C_5_H_8_O-CO]^+^	byakangelicol
21	7.322	262.1045	262.1041	C_5_H_17_O_8_N_4_	216.0965[M-CH_2_O_2_]^+^185.9843[M-H_12_O_2_N_2_]^+^	unknown
22	7.634	554.2458	554.2464	C_20_H_37_O_12_N_6_		unknown
23	7.910	246.2427	246.2420	C_13_H_31_ON_3_	228.2312[M-H_2_O]^+^	unknown
24	8.159	290.2686	290.2701	C_18_H_35_O	228.2320[M-C_3_H_4_]^+^	unknown
25	9.070	217.0466	217.0495	C_12_H_8_O_4_	202.0241 [M–CH_3_]^+^161.0598 [M–2CO]^+^	xanthotoxin
26	9.342	309.0731	309.0725	C_9_H_14_O_9_N_3_	224.0070[M-C_5_H_9_O]^+^	unknown
27	9.491	247.2754	247.0523	C_13_H_10_O_5_	232.0355 [M–CH_3_]^+^217.0120 [M-C_2_H_6_]^+^	pimpinellin
28	9.663	217.0466	217.0495	C_12_H_8_O_4_	202.0267 [M–CH_3_]^+^174.0322 [M–CH_3_-CO]^+^	bergapten
29	9.932	339.0842	339.0844	C_11_H_18_O_6_N_7_	324.1187 [M–CH_3_]^+^178.0837 [M-C_5_H_11_O_6_]^+^	unknown
30	10.161	287.0914	287.0914	C_16_H_14_O_5_	203.0331 [M–C_5_H_8_O]^+^	oxypeucedanin
31	10.576	187.1734	187.0382	C_11_H_6_O_3_	159.1311 [M–CO]^+^144.0484 [M–COCH_3_]^+^131.0532 [M–2CO]^+^	Psoralen
32	10.800	271.0965	271.0965	C_16_H_14_O_4_	203.0314 [M–C_5_H_8_]^+^	imperatorin
33	11.117	245.1172	245.1093	C_15_H_16_O_3_		osthole
34	11.437	271.0965	271.0965	C_16_H_14_O_4_	203.0336[M–C_5_H_8_]^+^159.0388[M–C_5_H_8_-CO_2_]^+^147.0438[M–C_5_H_8_-2CO]^+^	isoimperatorin

**Table 3:** Peak areas of thirty-four compounds from 12 batches of the YZT samples.

Peak	S01	S02	S03	S04	S05	S06	S07	S08	S09	S10	S11	S12
1	10244554	16930564	418890	332414	3942666	2059990	1120655	747919	13546055	13744838	12372915	3136280
2	7949749	9987599	1142314	3660481	4688970	697435	1138436	9399389	9384576	10125533	10152124	2481292
3	33700312	64824276	6702990	4053466	23353294	14888894	5252401	3076464	47050576	44901664	48581228	16425594
4	20469648	40011020	2216500	1357947	12035808	5985759	3238425	2274434	28107396	32772130	29441340	8782729
5	14380318	28329194	1026548	577282	5900520	2714479	1842725	790575	20550076	25968522	19358266	4767493
6	36856000	49879568	6847007	5696668	19740582	15763828	7561199	7564052	40185440	7564052	38231020	15571606
7	1711335	1485647	43194	36885	51993	130739	105094	52851	1041068	1414135	1211229	119994
8	3141931	3144220	677318	392959	2028766	1312728	589205	555387	3361033	3967358	3351850	1316145
9	42390400	86508888	37123712	37993380	32002554	19199566	33098436	70989704	62385824	70989704	61280180	20896936
10	34411664	49026132	4849252	3627606	17187142	13540835	5551001	5015446	39142264	5015446	37562304	14107432
11	18072188	28182211	1907938	1156952	9429763	4333017	2708047	2201167	25002894	26904977	29305297	5831961
12	14158135	26900244	1017588	1339987	8577751	6076479	3928110	1432471	17481408	1432471	18776652	10838332
13	4342307	11132227	971613	961612	2429294	1724858	932988	602764	6139924	4985228	7944996	1544894
14	41778884	113993560	7765805	7637866	30821784	18377190	12713581	6182978	63463984	6182978	75402024	19333384
15	23233662	37089924	4725501	5177430	6620499	4969553	4077647	8255664	27540176	8255664	24340236	8579587
16	5621262	12140419	367642	395094	1588666	1836682	828854	480598	8126569	13546001	7531148	2331528
17	88199904	139792256	10045018	10266522	23592612	18170168	12545877	7594058	109628488	178904080	97302768	31050786
18	3251394	3146738	2411079	2045014	3143703	3222117	1025955	2023451	4407905	4853269	3408821	2731474
19	7315382	10017907	5497550	5117611	6178704	7298941	3572644	6402335	11615372	6402335	7653924	7210558
20	1014255	2033594	603116	519644	1055068	1236775	296196	754212	2212803	2116750	1141295	1131468
21	199552	247769	132312	202473	138866	117894	176839	46747	208143	159810	262658	90720
22	148469	213096	436390	558607	165571	93589	97470	104341	1313237	1392525	180031	156080
23	1307923	981091	1694080	1082664	2096133	2586007	2002152	1393343	1661914	2382745	1933567	2251526
24	733420	525921	1150362	651512	1311777	1636515	1152949	817735	1184827	2074517	1169948	1472406
25	138868	315592	176341	151362	398823	1338634	514746	184070	157253	614760	211309	472431
26	510856	472611	404223	333423	463197	524719	199312	392701	721076	798630	575375	380331
27	8481836	5295768	7879050	6185282	10275968	11548586	10396492	7725108	14744180	21124483	9824059	11315813
28	284307	525066	256318	210104	308832	952450	348915	145043	168709	635424	352302	340595
29	1525608	1662515	1397379	1233607	1593201	1442143	723853	1111994	2362815	207924	1916669	1277259
30	77103	164981	44375	30012	83877	41054	26173	15009	169819	176342	91166	26842
31	2047545	1256449	884526	1895731	934015	1890348	2064941	1847992	5799667	7081346	790821	2048515
32	472899	1280813	3653542	3587152	5618072	5186924	2679041	269755	4937185	1272774	709036	5348282
33	8556	19603	10461	7867	10796	5508	11759	5362	10754	16191	8434	12334
34	7755	36081	21122	21213	19115	21704	16501	6598	20022	20369	17372	18464

Eighteen constituents were identified by comparing their chromatographs and TOF-MS results with those of standards. These constituents are as follows: protopine (6), tetrahydropalmatine (9), α-allocryptopine (10), coptisine (12), tetrahydroberberine (13), corydaline (14), palmatine (15), berberine (16), byakangelicin (19), byakangelicol (20), xanthotoxin (25), pimpinellin (27), bergapten (28), oxypeucedanin (30), psoralen (31), imperatorin (32), osthole (33), and isoimperatorin (34). Among these, 3 compound pairs, peaks 9 and 10, 13 and 14, and 19 and 20, had almost the same retention time, but they were resulted from 2 separate constituents, based on the MS information for standard compounds. Three overlapping peaks (RT: 4.715, 5.495, and 7.050) were identified as 2 independent constituents by comparison to the chromatogram of DL-tetrahydropalmatine and α-allocryptopine, tetrahydroberberine and corydaline, byakangelicin and byakangelicol, respectively. Peaks 2, 3, 4, 5, 11, 17 were tentatively identified by comparison with MS data from related literature [25]–[27] and the details were listed in **Table 2**. However, the identity of this constituent could not be confirmed because we were unable to obtain a standard for comparison. In a previous study, we found 17 common peaks using UV measurements and 15 of these were identified in the fingerprint analysis of YZT. However, because we used a method with high sensitivity and resolution to directly analyze intestinally absorbed YZT solutions, we found 34 constituents and identified 8 more compounds in the present study than previous research.

### 5. Chemical assessment of the intestinally absorbed YZT solutions from 12 YZT samples using hierarchical cluster analysis

**Figure 2:**
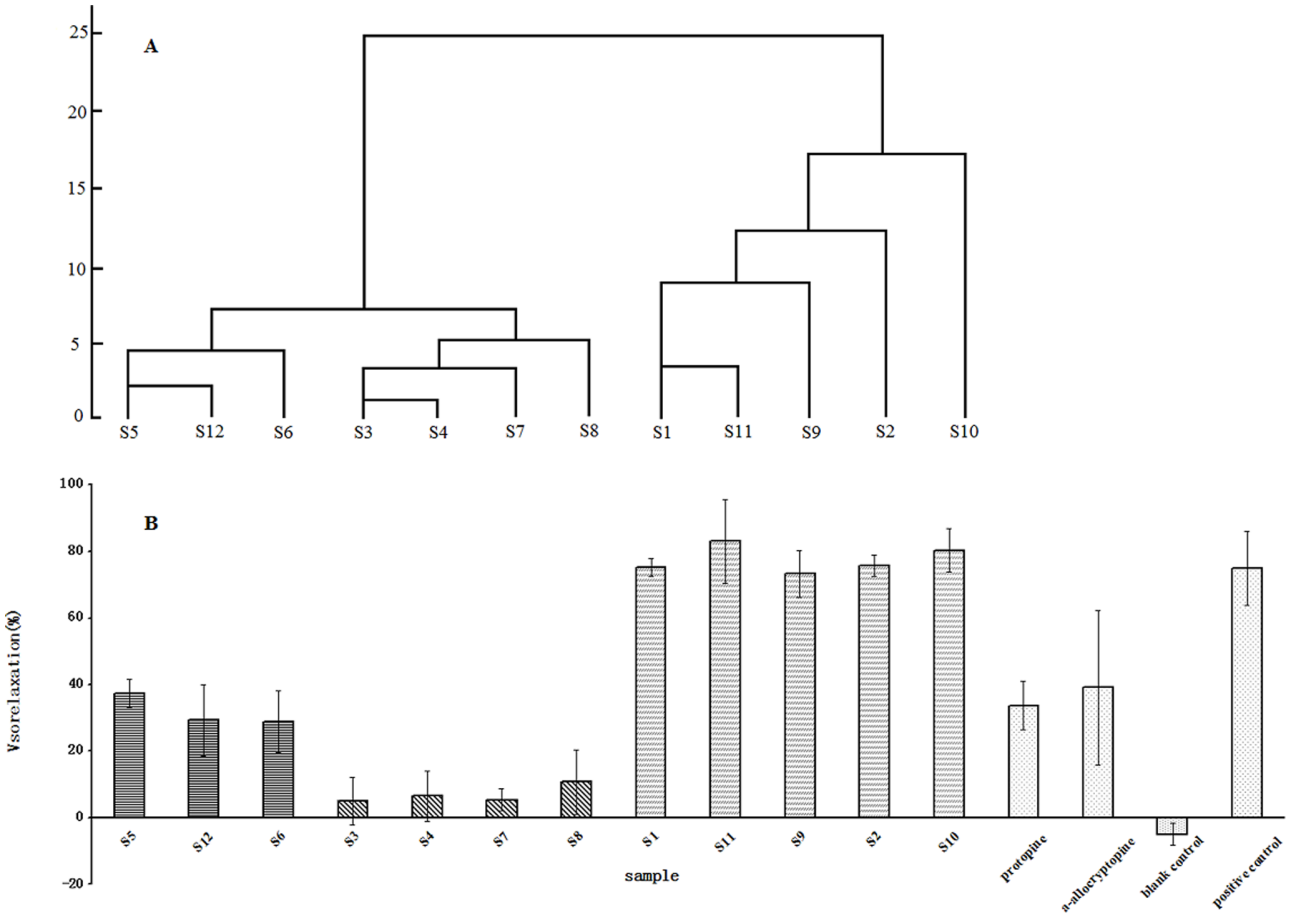
Chemical and bioactive profiles of the intestinally absorbed YZT solutions from 12 YZT samples. (A) Dendrograms of the Hierarchical Cluster Analysis based on the characteristics of 34 peaks area. (B) Vasorelaxation evaluation of the intestinally absorbed 12 YZT solutions, protopine, α-allocryptopine, blank and positive control using KCl-precontracted rat aortic rings (n = 8).

As can be seen in **Figure 2 (A)**, samples 5, 6 and 12 from Manufacturer C, samples 3, and 4 from Manufacturer B in Guangxi, and samples 7 and 8 from a pharmaceutical factory in Chongqing were first aggregated into 2 respective groups and were then aggregated together. Similarly, samples 1 and 2 from Manufacturer A and samples 9, 10, and 11 from Manufacturer F were included in the same group. The results indicated that chemical profiles of YZT obtained from the same factory were similar, whereas the chemical profiles of YZT obtained from different factories were significantly different, as which had been found previously [18], [19]. However, it is difficult to judge the quality of CMM products solely from chemical profiles, including chemical fingerprints and the multi-marker constituents of CMM formulations.

### 6. Vasorelaxation evaluation

Endothelial dysfunction was a very important reason for headache (chronic migraine) [28] and dysmenorrheal [29]. At present, many data [30]–[32] suggested that chronic migraine and cardiovascular disease had the same pathophysiological mechanisms as follows: abnormal control of systemic vascular tone, alterations in systemic arterial structure and function and impaired ability to repair systemic endothelial injury. Moreover, there existed potential relationship between the presence of migraine and congenital heart defects [33]. Meanwhile, our previous research indicated that YZT was predicted to possess the pharmacological action of calcium channel receptors *in silico* which can play a crucial role in acute endothelium-dependent vasodilator responses to contract smooth muscle of the blood vessels [34]. Thus, both vasorelaxation of aortic rings and basilar artery should be evaluated systemically in order to shed light on YZT samples curing chronic migraine. However, vasorelaxation of aortic rings were performed using intestinally absorbed YZT solutions and the results showed that this model in vitro exhibited very stable and sensitive in the previous research [5].

Preliminary experiments were performed to evaluate the experimental conditions of the *in vitro* vasorelaxation method and to determine the stability of samples during testing. Different concentrations of intestinally absorbed YZT solutions and different incubation times were investigated for the intestinal absorption coupled with bioactivity experiment. A dose-dependent vasorelaxant effect was produced using intestinally absorbed YZT solutions at low, middle, and high concentrations and besides, 2 h of incubation was required to reach maximal vasorelaxation [5]. In addition, intestinally absorbed YZT solutions of 6 cumulative doses were investigated and 1600 mL of the intestinally absorbed solution was selected as the maximum dose for additional experiments. Ligustrazine is an active constituent of Ligusticum chuanxiong Hort (Umbelliferae) and it has been known as a vasorelaxation compound [35], so ligustrazine was chose as a positive control in the pharmacological tests, which had exhibited very good vasorelaxation (74.96±11.10) in this study.

Blood vessel activities *in vitro* were evaluated for YZT of 12 batches, the blank control and the positive control and the results were shown in **Figure 2 (B)**. All samples of YZT and the positive control possessed evident blood vessel activities (BVC) in contrast with the blank contol. However, BVC activity differed between batches of YZT. The maximum BVC was 83.08±12.66 and was obtained using S11, and minimum BVC was 5.11±7.17 and was obtained using S3. However, in order to distinguish YZT samples from the positive control, chemical profiles were needed to be analyzed. Fortunately, the results suggested that samples grouped by HCA from chemical profiles have similar bioactivity while samples in different groups from chemical profiles displayed very different bioactivity. Thus, the use of a combinative bioassay that includes an intestinal absorption test coupled with an *in vitro* bioactivity experiment may increase the level of CMM quality control from both chemical and bioactive profiles.

### 7. Bridging the relationship between constituents and vasorelaxation

Using a polynomial fitting technique, we established a mathematical model to characterize the correlation between peak areas of chemical constituents of YZT and vasorelaxation. The computational simulation showed that the results from our model are fited well with the experimental data. To obtain this model, we constructed a transformation 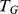 (see Eq. 5 in Section 3.7) based on GRA. 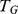 was used to translate the 34-dimension raw sample data set into a 1-dimension data set, so that a single variable fitting technique could be used to solve this multivariable mathematical modeling problem. There are no related studies reporting the use of this transformation.

In order to obtain the mathematical model that establishes the correlation between constituents of YZT and vasorelaxation, the following transformation based on GRA was introduced: 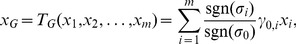
(4)where 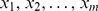 are the peak areas of chemical constituents of YZT.

Based on **Table 4** and (4), the following equation was obtained.
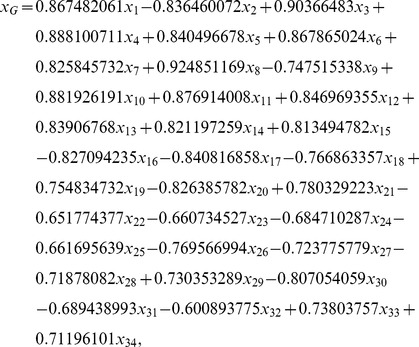
(5)


**Table 4:** GRG, GRP and the standard deviation (SD).

peak number *i*	GRG *γ*_0,*i*_	SD
1	(+) 0.86748	0.09537
2	(−) 0.83646	0.1334
3	(+) 0.90366	0.09936
4	(+) 0.88810	0.10255
5	(+) 0.84050	0.11336
6	(+) 0.86787	0.14523
7	(+) 0.82585	0.1483
8	(+) 0.92485	0.05456
9	(−) 0.74752	0.12483
10	(+) 0.88193	0.16877
11	(+) 0.87691	0.07082
12	(+) 0.84697	0.18052
13	(+) 0.83907	0.12834
14	(+) 0.82120	0.1837
15	(+) 0.81349	0.13422
16	(−) 0.82709	0.14227
17	(−) 0.84082	0.12528
18	(−) 0.76686	0.09382
19	(+) 0.75483	0.12724
20	(−) 0.82639	0.12968
21	(+) 0.78033	0.15595
22	(−) 0.65177	0.18974
23	(−) 0.66073	0.08499
24	(−) 0.68471	0.1245
25	(−) 0.66170	0.18922
26	(−) 0.76957	0.11292
27	(−) 0.72378	0.12888
28	(−) 0.71878	0.1709
29	(+) 0.73035	0.13824
30	(−) 0.80705	0.09864
31	(−) 0.68944	0.14459
32	(−) 0.60089	0.16733
33	(+) 0.73804	0.14255
34	(+) 0.71196	0.14255

(+) and (−) represent 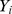 and 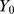 are positive relation and negative relation, respectively.

Samples 01–10 were used to establish the model. Using (5) and a polynomial fitting technique, the following mathematical model was obtained:
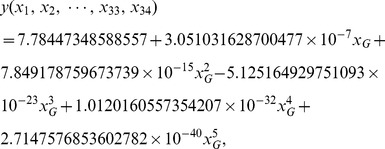
(6)where 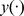 is the degree of vasorelaxation. As can be seen from **Table 5**, the average bias ratio of modeled values to experimental data was 9.81% and 6 of the bias ratios (60%) were less than 10%.

**Table 5:** Comparison of experimental data and modeled values.

Sample NO.	Experimental Data [%]	Modeled Values [%]	Bias Ratios [%]	Average Bias Ratio [%]
#S01	75.15	67.81	9.77	
#S02	75.78	75.72	0.08	
#S03	5.11	5.33	4.31	
#S04	6.47	5.22	19.32	
#S05	37.28	44.75	20.04	9.81
#S06	28.83	24.64	14.53	
#S07	5.37	6.33	17.88	
#S08	10.76	11.54	7.25	
#S09	73.19	76.71	4.81	
#S10	80.24	80.15	0.12	
*S11	83.08	82.84	0.29	4.16
*S12	29.28	26.93	8.03	

^#^, used to perform mathematical modeling;

^*^, used to be model validation.

In addition, samples 11 and 12 were used for assay validation. Validation results were shown in **Table 5**. Table 5 indicated that the average bias ratio of modeled values to validation experimental data was 4.16%, and all bias ratios were less than 10%. Therefore, validation experimental data supported the theoretical predictions of the model (6). Furthermore, we could predict vasorelaxation effects of YZT by determining the peak areas of YZT constituents. From a practical point of view, this model could help us to develop software that was capable of computing the degree of expected vasorelaxation from the peak areas YZT of constituents.

### 8. Identifying key components of YZT for vasorelaxation

Because only 12 samples were analyzed, general statistical analysis methods were unavailable not for data analysis. However, GRA, on the other hand, is suitable for analysis of data from “small sample sizes,” and can achieve good results when evaluating with “poor information”, and of uncertain systems. Thus, GRA was used to identify key components of YZT for vasorelaxation. The results of the GRA were shown in **Table 4** (in Eq. 2, the value of 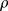 is 0.5). GRG and relational polarity analysis indicated that there were 18 components of YZT that have had increase vasorelaxation. In addition, these components could be ranked from high to low in terms of their effect on vasorelaxation as follows:
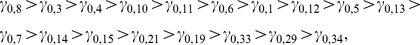



Key constituents were identified by the value of GRG 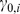 which could reflect each compound for the extent of influence on vasorelaxation. Shown in **Table 4**, the GRG 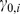 values of compound 3 and 8 were greater than 0.90, which suggested that the two components had marked influence. At the same time, the GRG 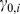 values of compound 1, 4, 6, 10 and 11 were at the range from 0.85 to 0.90, suggesting that the five compositions had a relatively high influence on vasorelaxation. Therefore, the seven constituents were considered as key components which could play very important roles on bioactivities. Unfortunately, only compound 6 and 10 were identified as α-allocryptopine and protopine by the standards and compound 11 were tentatively identified by related literature. The other constituents were unknown because of lacking of correlative standards and literatures. Thus, the bioactivities of key constituents (compound 6 and 10) were validated and the results suggested that α-allocryptopine and protopine exhibited very obvious vasorelaxation of 33.66±7.13 and 38.97±23.23, respectively, as shown in **Figure 2 (B)**. However, quantitative analysis of seventeen constituents in previous studies [19] could not control the quality of YZT samples because of lacking of quantitative information of several key constituents for bioactivity. Therefore, a series of chemical separation, structure identification and quantitative analysis of these key compositions should be carried out further research.

### Conclusions

CMM, as a complexity system, has been confronted with great difficulties for the modernization of traditional Chinese medicine. Among them, bridging RCB and identifying KC were one (s) of the critical problems, which could increase the level of the quality control, improve the efficacy and reduce the toxicity of CMMs. In this work, the combinative bioassay *in vitro*, intestinal absorption test coupled with the bioactivity experiment, was developed to probe active constituents and their synergistic mechanism of YZT in vasorelaxation. The results indicated that chemical profiles combining with bioassay assessment, could evidently increase the level of quality control for improving the efficiency of CMMs. Main active compounds were identified and might be used as marker substances of quality control of YZT. At the same time, the correlation between constituents of YZT and vasorelaxation was established based on the mathematical model of GRA. The results suggested that it could utilize peak areas of constituents of YZT to forecast the values of vasorelaxation by the establishing mathematical model. However, many CMMs should undergo the gut flora biotransformation and the hepatic metabolism to produce some metabolites which were likely to be bioactive constituents *in vivo*. Therefore, more complex model including gut flora biotransformation, intestinal absorption and hepatic metabolism *in vitro* should be developed to probe the active constituents of CMMs. At the same time, in order to appraise the bioactivities of YZT samples systemically, vasorelaxation of basilar artery should be further performed in the future.

Our main highlight findings are as follows:

1. The combinative bioassay, an intestinal absorption test coupled with an *in vitro* bioactivity experiment, was developed to be a simple, sensitive, and stable pharmacological method for evaluating CMM formulations. This bioassay allows for the exclusion of unabsorbed constituents that might interfere with the *in vitro* bioactivity and can be directly applied to evaluation of CMM product quality.
2. Chemical analysis of intestinally absorbed solutions of 12 YZT samples was carried out using RRLC-Q-TOF. The high sensitivity and resolution of the RRLC-Q-TOF method resulted in the identification of 34 constituents common to all samples. The number of constituents identified in this study is obviously greater than the number previously reported for YZT chemical fingerprints [19].
3. A mathematical model was established to bridge the correlation between the YZT chemical constituents and vasorelaxation. Because we had a small sample size, we used GRA to analyze our results. A single-variable fitting technique was used to solve the multivariable mathematical modeling problem. The model developed as part of this study can be used to develop software that can compute the expected degree of vasorelaxation from the peak areas on chromatographs of YZT constituents.

## Supporting Information

Figure S1 **RRLC-ESI-Q/TOF chromatograms (TIC) of the intestinally absorbed YZT solutions from 12 YZT samples.** (A) sample 1, (B) sample 2, (C) sample 3, (D) sample 4, (E) sample 5, (F) sample 6, (G) sample 7, (H) sample 8, (I) sample 9, (J) sample10, (K) sample 11, (L) sample 12.
(TIF)
